# Mechanism and Impact of Digital Economy on Urban Economic Resilience under the Carbon Emission Scenarios: Evidence from China’s Urban Development

**DOI:** 10.3390/ijerph20054454

**Published:** 2023-03-02

**Authors:** Songtao He, Shuigen Yang, Amar Razzaq, Sahar Erfanian, Azhar Abbas

**Affiliations:** 1 School of Economics and Trade, Hunan University of Technology and Business, Changsha 410205, China; 2 Business School, Huanggang Normal University, Huanggang 438000, China; 3 Institute of Agricultural and Resource Economics, University of Agriculture, Faisalabad 38040, Pakistan

**Keywords:** efficiency, climate sustainability, urban development, quantitative assessment

## Abstract

China is currently experiencing a phase of high-quality development, and fostering the resilience of the urban economy is key to promoting this development. The growth of the digital economy is seen as critical to achieving this goal. Therefore, it is necessary to study the mechanism by which the digital economy affects urban economic resilience and the impact of carbon emissions. To this end, this paper empirically analyzes the mechanisms and impacts of the digital economy on urban economic resilience using panel data from 258 prefecture-level cities in China between 2004 and 2017. The study employs a two-way fixed effect model and a moderated mediation model. The results show that: (1) The development of the digital economy can significantly improve the resilience of the urban economy in different periods and different city sizes; (2) The development of the digital economy promotes the economic resilience of developed cities and eastern cities more significantly; (3) In the context of carbon emissions, the digital economy positively contributes to urban economic resilience through population quality and industrial structure but negatively contributes to urban economic resilience through above-scale enterprises; (4) Carbon emissions have a positive moderation effect on the historical path of the industrial structure, above-scale enterprises, and the front path of population quality in the mechanism of the role of the digital economy on the economic resilience of cities, and a negative moderation effect on the front path of above-scale enterprises. Based on these findings this paper proposes several suggestions, such as revolutionizing the digital development of cities, optimizing regional industrial collaboration, accelerating the training of digital talents, and preventing the disorderly expansion of capital.

## 1. Introduction

Resilience refers to the ability of a system, community or society exposed to a disaster to withstand, absorb, accommodate, adapt, transform, and recover from the impact of a disaster in a timely and effective manner [1]. In recent years, the resilience performance of cities in the fields of ecological environment, climate change, disaster risk, and social economy has attracted great interest from the academic community. In 2012, UNDRR launched the “How to Make Cities More Resilient” campaign [2]. Moreover, in 2013, the World Bank developed a “Guide to Building Resilience in East Asia” [3]. Furthermore, in 2021, for the first time, the “Outline of the People’s Republic of China 14th Five-Year Plan for National Economic and Social Development and Long-Range Objectives for 2035” put forward the idea of building resilient cities [4]. Economic resilience, as one of the significant aspects of urban resilience [5], is the key to a city’s ability to withstand risks, recover its economy, and adapt to adjustments after an economic shock [6]. Given, China’s experience in coping with several external shocks, such as the Asian Financial Turmoil, SARS, the US Sub-prime Crisis, and COVID-19, it has become increasingly clear that economic resilience plays a crucial role. Thus, promoting urban economic resilience scientifically is the top priority for seizing the initiative in economic development and preventing and defusing major crises.

Improving the prevention of risk shocks and enhancing the resilience of urban economy can be achieved through various means such as realizing industrial diversification, enriching communication network, improving the industrial chain, digitizing geographic information, and securing development practices [7]. One of the fundamental pillars of the digital economy, digital technology has facilitated the enrichment of communication networks, the digitization of geographic information, and the security of development practices. Studies have shown that the digital economy plays a significant role in economic growth, structural adjustment, and innovative development [8]. In 2015, 193 member states of the United Nations affirmed the significant contribution of information and communication technology to accelerating human progress, bridging the digital divide, and developing scientific and technological knowledge. Moreover, the UN recognized the digital economy as a key driver for sustainable economic development [9]. Sustainable development comprises economic sustainability, ecological sustainability, and social sustainability, all of which are integrated with urban economic resilience [10,11].

Notably, China has made significant strides in the digital economy in recent years, and this has played a key role in driving economic development. According to the Global Digital Economy White Paper, in 2020, the scale of Chinese digital economy was 5.4 trillion US dollars, ranking second in the world, and with a year-on-year growth rate of 9.6%, ranking first in the world. Remarkably, China achieved breakthroughs in both scale and growth despite the pandemic [12]. Thus, promoting the development of the digital economy is crucial for building a solid economic and technological foundation for cities. It also provides a significant guarantee for cities to resist economic risks, restore economic strength, and adjust economic development accordingly.

Moreover, the digital economy can also help cities to achieve their sustainability goals, particularly with respect to reducing carbon emissions, which can serve as an indicator of the city’s potential resilience [13]. On the one hand, the digital economy serves as a green manufacturing method that optimizes industrial output and organizational decision-making [14]. Specifically, digital technology can improve production processes and reduce energy consumption, monitor energy consumption in real-time, and promote green industries such as shared and smart transportation, which can encourage a low-carbon lifestyle. On the other hand, carbon emissions are closely linked to energy structure and environmental conditions, which are key determinants of economic resilience [15]. Energy structure affects energy security and sustainability, while environmental conditions directly affect the quality of life and health of urban residents, both of which can have negative impacts on the city’s economic activity. Furthermore, in 2020, China set the ambitious strategic goals of “carbon peaking” and “carbon neutrality”, necessitating a more comprehensive examination of the relationship between the digital economy, carbon emissions, and urban economic resilience. In the context of the digital economy, such an examination can provide a theoretical foundation for cities to achieve sustainable development and carbon neutrality.

Against this backdrop, this paper seeks to examine the relationship between the digital economy, carbon emissions, and urban economic resilience in China. In particular, the paper aims to explore the measurements of digital economy and urban economic resilience, the heterogenous manifestations of the digital economy on urban economic resilience in different cities, the identification of the impacts and mechanisms of the digital carbon emissions and urban economic resilience, and the role of carbon emissions in moderating the relationship between two. The discussion on these issues can contribute to the synergistic development of digital economy and urban economic resilience in China and enrich empirical research on the relationship between the two.

## 2. Literature Review

The term “resilience” originates from “engineering resilience”, which refers to describe the scalability of matter or body. Ecological researcher Holling introduced the concept of “resilience” into ecological research [16], which was later refined and expanded to include evolutionary resilience and economic resilience. In fact, economic resilience has its roots in evolutionary resilience [17], which emphasizes the ability to solve problems and achieve stable long-term development. 

Urban economic resilience refers to an area’s ability to quickly recover, or return to its previous level of employment and output, following an external shock. The academic community’s understanding of economic resilience, including its connotation, measurement, and empirical study, has increasingly become comprehensive. Scholars have defined economic resilience from four dimensions: resistance, recovery, adjustment and evolution [18], and this definition has gained widespread accepted [19,20]. 

In terms of measurement research, scholar have used various evaluation methods for economic resilience, including the single and double core variable method [21,22] and index system method [23]. In theoretical and empirical research, scholars have primarily focused factors such as industrial structure [24,25], social capital [26,27], policies and institutions [28], and cultural factors [29] to discuss urban economic resilience at different scales, including districts, cities, provinces, countries, and specific regions.

The concept of “digital economy” has undergone a series of transformations since its first introduction by Tapscott in 1996 [30], evolving from the information economy to the internet economy and, eventually, the new economy [31]. The G20 Summit in Hangzhou in 2016 played a significant role in shaping the commonly accepted definition of the digital economy. This definition emphasizes the use of digitized information and knowledge as the key factor of production, modern information networks as a critical activity space, and the effective use of information and communication technology (ICT) as a driving force behind productivity growth and economic structural optimization. Digital economy leverages new digital technologies such as the Internet, cloud computing, big data, Internet of Things (IoT), and fintech to collect, store, analyze, and share information digitally and transform social interactions. As a result, modern economic activities are becoming more flexible, agile and intelligent [32].

However, despite the widespread acceptance of the term, the conceptual connotation of digital economy remains a topic of debate. National economic accounting [33], calculation of added value [34], compilation of relevant indexes [35], and construction of satellite accounts [36] are the primary methods for evaluating the digital economy. Scholars have mainly focused their theoretical and empirical research on the characteristics, effects, and the development path of the digital economy. For instance, Song et al. used the mediation effect model and the spatial Durbin model to explore the transmission mechanism and regional heterogeneity of the impact of the digital economy on ecological well-being performance [37]. Meanwhile, based on panel data of 277 cities in China from 2011 to 2018, Li et al. explored the impact mechanism and regional heterogeneity of the digital economy on the efficiency of the green economy [38].

To build on the topic of digital economy and urban economic resilience, it is important to note that while some research has been conducted on the impact of digital economy on urban economic resilience, there is still a lack of comprehensive studies exploring the relationship between the two. Linbo [39] explored the theoretical impact of digital economy on urban economic resilience, while Yan [40] and Jinhe [41] investigated the mediating mechanism and spatial effect of digital economy on urban economic resilience at the economic regional and national levels, respectively. However, the integration of the digital economy, carbon emissions, and urban economic resilience in the same framework, as well as a deeper exploration of the mechanism of the impact of the digital economy on urban economic resilience, has not been extensively studied. To fill this gap, this study utilizes data on 258 prefecture-level cities in China from 2004 to 2017 and constructs a two-way fixed effect model to examine the direct impact of digital economy on urban economic resilience. Furthermore, the study explores the heterogeneity of the impact of digital economy on the economic resilience of different cities and uses instrumental variables, replaces control variables, and adds dummy variables for robustness testing. Finally, mediation variables such as industrial structure, population quality, above-scale enterprises are selected, and a moderated mediation model is constructed to investigate the internal mechanism of the impact of digital economy on urban economic resilience. Through these analyses, this research aims to provide a deeper understanding of the relationship between digital economy and urban economic resilience.

## 3. Theoretical Model

**Figure 1:**
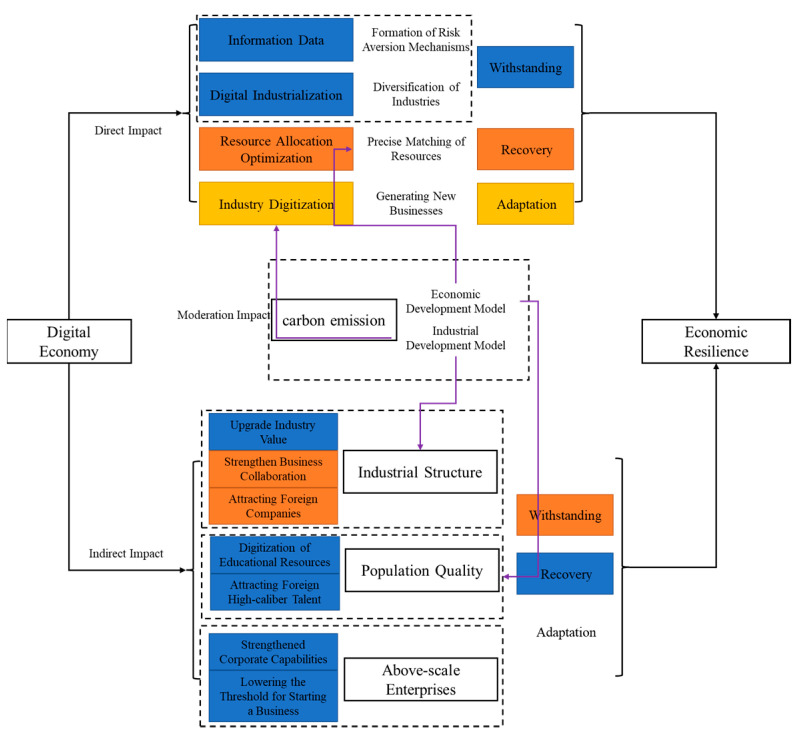
Pathway illustrating the impact of digital economy on urban economic resilience.

The concept of urban economic resilience encompasses resistance and recovery, adaptation and adjustment, innovation and transformation. On the other hand, the digital economy is characterized by quickness, directness, high permeability, external economy, sustainability and increasing marginal revenue. As a new engine to enhance urban economic resilience, the digital economy directly affects the urban economic resilience. The indirectly effects of the digital economy on urban economic resilience are mediated by factors such as industrial structure, population quality and scale enterprises, as illustrated in Figure 1.

### 3.1. Direct Impact Mechanism of Digital Economy on Urban Economic Resilience

As previously mentioned, urban economic resilience is composed of three aspects: resistance and recovery, adaptation and adjustment, and innovation and transformation. Changes in capacities of urban resistance, recovery and adaptation can significantly affect the level of urban economic resilience. Although digital facilities and construction may exacerbate urban energy consumption and digital challenges such as cyber-attacks and fake news, they can still enhance the economic resilience of cities.

In terms of resistance, digital technologies such as the internet can digitize production information, business information, geographic information, and supply and demand information, thereby enabling prediction of risks and the development of better risk avoidance mechanism. By increasing the amount of information data available, the city’s ability to resist risks improves, and ultimately the city’s economic resilience is enhanced. At the macro level, the development of digital industries such as artificial intelligence, big data, blockchain, and cloud computing has fostered diversified industrial development in cities.

In terms of recovery, the digital economy plays an essential role in optimizing the allocation of urban resources [42]. The digital economy can facilitate the management of the matching, communication, investment, and circulation of tangible and intangible assets through digital means, thus enhancing the allocation of urban resources. As a result, during a crisis, cities can efficiently allocate and accurately connect resources, thereby improving their recovery ability and ultimately enhancing their economic resilience.

Regarding adaptability, the digital economy has spawned new formats and models of urban industries [43]. The combination of digital economy, production and life has promoted the development of novel formats and models, including online consumption, online teaching, off-site office, smart travel, and unmanned stores, which provide alternatives for urban economic operations. During COVID-19, the importance of digital technology such as the internet became even more evident as countries such as China adjusted their production and life patterns and vigorously promoted the counter trend growth in economy through adoption of cloud teaching, cloud office, cloud consumption, cloud tourism and other digital means. These adjustments have driven the reverse growth of the urban economy and improved the cities’ ability to adjust and adapt. 

Building upon the aforementioned points, we propose the following hypothesis:

**H1:**  *Digital economy has a direct and significant impact on promoting urban economic resilience.*

### 3.2. The Indirect Effect Mechanism of Digital Economy on Urban Resilience

#### 3.2.1. Industrial Structure

The integrity of the industrial chain and the improvement of the level of basic industry development are crucial to the economic resilience of cities. Although the digital economy has some adverse effects on the industrial structure, such as accelerating the decline of traditional industries, it has an overall positive impact on economic resilience. A complete industrial chain with deep integration and the strong synchronization can unite to resist risks [44], while a higher level of development of basic industries provides greater support for repairing other industrial chains and enhancing economic potential.

Improving economic resilience of cities requires supporting the industrial chain to resist risks and recover the economy. Addressing weak links in the chain, strengthening cooperation among enterprises, and upgrading the value of industries can improve the industrial chain. The development of the digital economy greatly increases its attractiveness to enterprises, accelerating the expansion of the urban digital economy and increasing its scale, scope and marginal revenue. The location advantages and development opportunities provided by the digital economy attract more potential enterprises to integrate into the city. 

Digital technology is also an important method to improve industrial cooperation, [45], strengthening the connection and cooperation between enterprises and upstream-downstream-enterprises, and providing an important guarantee for accelerating industrial collaboration, and promoting industrial integration and development. Additionally, digitization is of great significance for upgrading industrial value, as it leverages digital technology to promote technological innovation in traditional industries. In conclusion, the industrial chain plays a critical role in enhancing economic resilience, and the digital economy can indirectly improve urban economic resilience by stabilizing the industrial structure. Based on this, we propose the following:

**H2:**  *Digital economy indirectly improves the urban economic resilience by stabilizing industrial structure.*

#### 3.2.2. Population Quality

High-quality human resources are essential for post-disaster reconstruction in cities, and their impact on urban economic resilience primarily stems from their working and consumption abilities. First, high-quality human resources enable a city’s ability to withstand risks, recover, and innovate in response to urban problems [37]. Thus, in the event of an economic shock, high-quality human resources are crucial to forming an orderly post-disaster resilience model that improves the city’s economic resilience. Second, population resources are vital for alleviating production capacity pressures [46]. In times of economic crisis, addressing production capacity shortfalls requires a significant amount of manpower to carry out remedial measures and meet urban life needs. Conversely, overcapacity requires more consumers and stronger purchasing power to reduce consumption pressures, relieve the pressure on production capacity and maintain economic stability.

To increase the number of high-quality talent, two primary methods are used: attracting foreign high-quality talent and improving the quality of the local population. The digital economy has significantly reduced the cost of urban education, popularized educational opportunities, and improved the quality of education through the digitization of educational resources [47]. Thus, the digital economy plays a vital role in improving the quality of urban population. Second, the development of the digital economy increases the attraction of high-quality talents from abroad. The integration of digital economy, urban public management and service industry expands the overall welfare level of the city, providing greater urban happiness that attracts more potential high-quality talents to migrate to the city.

Based on this, a hypothesis is proposed as follows:

**H3:**  *Digital economy indirectly improves urban economic resilience by enhancing population quality.*

#### 3.2.3. Scale Enterprise

Scale enterprises play an important role in the recovery and development of the urban economy after facing distress. These enterprises, as the key components of the industrial chain, serve as the core of the organizational structure. The more scale enterprises and the stronger their drive, the more efficient the recovery or reconstruction of the industrial cluster relationships will be, thus helping to restore small and medium-sized enterprises and drive the restoration of the entire industrial chain.

Determining the number of enterprises above a designated size is based on the scale and number of enterprises. The digital economy, with its emerging technologies such as artificial intelligence, blockchain, and the Internet of Things, has broken through the traditional space-time constraints, enabling inter-regional information transmission and economic exchanges to be carried out on a smaller time scale and a larger spatial scale. This strength of the digital economy can strengthen the innovation capabilities of enterprises [48] and promote more enterprises to grow into scale enterprises. Moreover, it is important to note that the diffusion of the digital economy is not neutral, and can trigger the Matthew effect among enterprises. This effect is characterized by a dynamic that alters the flow of information and the distribution of rewards in ways that favor high-status actors, resulting in the accumulation of advantages for them and creating a two-tier division, ultimately leading to the growth of larger enterprises.

Regarding the number of enterprises, the digital economy, with its big data resources, has improved the integration efficiency of market information resources and reduced the probability of resource misallocation. This digital economy can reduce the entrepreneurial risks and costs of small and medium-sized enterprises, increase the base of entrepreneurial enterprises, and then realize economic growth. By reducing the entrepreneurial risks and costs of small and medium-sized enterprises, the digital economy can realize the diversified development of “mass entrepreneurship and innovation”, and provide more possibilities for the types and number of scale enterprises.

Based on the above discussion, the following hypothesis is proposed:

**H4:**  *The digital economy indirectly improves urban economic resilience by increasing the number of above-scale enterprises.*

### 3.3. The Moderating Effect of the Digital Economy on Urban Economic Resilience

Carbon emissions serve as an indicator of urban economic and industrial development models [49], with high emissions reflecting a more extensive and simple model and low emissions indicating a more intensive and diversified model. However, the economic and industrial development model can limit the potential of the digital economy in enhancing economic resilience. In terms of economic development, cities with extensive development have a strong dependence on energy resources and can harm the environment [50], which restricts the digital economy’s ability to optimize the resource allocation and attract high-quality talent, limiting its potential to improve the city’s resilience to risks and restore the economy. Similarly, a single industrial model in a city can impede the development of industrial convergence [51], and limit the emergence of new business formats and models, which can negatively affect the digital economy’s ability to promote innovation and improve the industrial chain, further impairing its capacity to enhance the city’s resilience to risks. Therefore, we propose that carbon emissions can moderate the role of digital economy in enhancing economic resilience. 

Based on the above discussion, we proposed following:

**H5:**  *Carbon emissions moderate the impact of digital economy on the economic resilience of cities.*

## 4. Materials and Methods

### 4.1. Empirical Model Construction

1. Benchmark regression model. To examine the overall impact of the digital economy on urban economic resilience and test hypotheses H1, a fixed-effect model is constructed as a benchmark regression model:

(1) $$RES_{it}=\beta_{0}+\beta_{1}DIG_{it}+\beta_{set}Z_{it}+\lambda_{1i}+\gamma_{1t}+\varepsilon_{1it}$$

where RES, DIG and Z, respectively, represent the urban economic resilience level of the explained variable, the digitization level of the explanatory variable, and control variable. ε is a random disturbance term, λ and γ express individual fixed effect and time fixed effect, respectively. i and t denote individual and time, respectively. $\beta_{1}$ represents the digital economy’s overall impact on economic resilience of cities.

2. Conditional process analysis [52]. In addition, to explore other mechanisms by which the digital economy affects the resilience of urban economies, the article draws on the research methods of Baron [53] and Wen Zhonglin [54,55] to construct a moderated mediation model combined with a benchmark regression model to examine the mediating and moderating effects of the digital economy on the resilience of urban economies, and to test hypotheses H2, H3, H4, H5.

(2) $$RES_{it}=c_{0}+c_{1}DIG_{it}+c_{2}CR_{it}+c_{3}CR_{it}DIG_{it}+c_{set}Z_{it}+\lambda_{2i}+\gamma_{2t}+\varepsilon_{2it}$$

(3) $$MED_{it}=a_{0}+a_{1}DIG_{it}+a_{2}CR_{it}+a_{3}CR_{it}DIG_{it}+a_{set}Z_{it}+\lambda_{3i}+\gamma_{3t}+\varepsilon_{3it}$$

(4) $$RES_{it}={c^{\prime }}_{0}+{c^{\prime }}_{1}DIG_{it}+{c^{\prime }}_{2}CR_{it}+{c^{\prime }}_{3}CR_{it}DIG_{it}+b_{1}MED_{it}+b_{2}CR_{it}MED_{it}+b_{set}Z_{it}+\lambda_{4i}+\gamma_{4t}+\varepsilon_{4it}$$

where MED represents three mediating variables: industrial structure, population quality and scale enterprises; CR represents carbon emissions;$\;a_{1}\;\mathrm{and}\;a_{3}$ respectively represent the effect of digital economy on the mediating variable;$\;\mathrm{and}\;b_{1}\;\mathrm{and}\;b_{2}$ respectively represents the effect of the intermediary variable on the explained variable. The remaining variables have the same meaning as those in Model 1.

### 4.2. Data Description

1. Explained variable. The explained variable in this study is economic resilience (RES), which is divided into three dimensions—resistance and recovery, adaptation and adjustment, innovation and transformation—based on Martin’s definition of regional economic resilience [17], and index selection from Jianjun [56], Jinhe [25], and Wu [57] (as shown in Table 1). The feasibility test showed that the PCA method is suitable for urban economic resilience, as the KMO and Bartlett spherical test were 0.881 and 0.00, respectively.

2. Explanatory variable: The explanatory variable is the digital economy (DIG), which is measured using a digital economy index system constructed by this paper based on Tao’s [58] work on digital infrastructure and digital industrialization (as shown in Table 1). The Topsis method is used to measure the level of the digital economy.

3. Mediating variables. The study also examines three mediating variables—industrial structure (IS), population quality) PQ), and scale enterprise (MC). According to Liu’s [60] research, the stability of an industrial structure is measured by the entropy of the industrial structures, where a smaller value indicates greater stability and the more balanced the development across primary, secondary, and tertiary industries. This stability provides a strong foundation for the cities to continue production activities and regulate production priorities even during difficult times. Population quality is measured by the number of students in general colleges and universities relative to the total population at the end of the year, as per Guangbin’s [61] research. A higher value indicates better quality of urban population, which serves as a reserve of human resources to resist risks and restore urban economy in times of crisis. (Scale enterprise (MC) is measured by the number of above-scale enterprises relative to the total population at the end of the year, where the quality of enterprises is vital to the market economy’s vitality. The quantity and quality of enterprises significantly influence the innovation behavior of the market, and consequently, the innovation and transformation ability of cities.

4. Moderating variables. The moderating variable in this study is the carbon emissions (CR), which is calculated using data from the county-level inventory of China’s carbon emission database [62]. This variable is used to measure the impact of the digital economy on urban economic resilience.

5. Control Variables. Four control variables are set in this study, including economic density (ED, GDP/land area of administrative region), population density (PD), economic openness (EO, amount of foreign capital actually used in the current year), and food security (FS, value-added of primary industry/total population at the end of the year). These variables reflect urban economic development, economic input, and the basic production and living security of a city.

The study uses panel data from 258 prefecture-level cities in China from 2004 to 2017. Date on carbon emission were obtained from the China Carbon Emission Accounts & Datasets (CEADs), and the innovation and entrepreneurship index were obtained from Center for Enterprise Research of Peking University. The rest of the data were obtained from China Urban Statistical Yearbooks, Provincial Statistical Yearbooks, some city statistical bulletins, China Customs, EPS database, and National Research Network. Missing values were dealt with using the mean method and the neighborhood method. Descriptive statistics of variables are shown in Table 2.

## 5. Results and Discussion

### 5.1. The Direct Impact of the Digital Economy on Urban Economic Resilience

#### 5.1.1. Benchmark Regression

To investigate the direct impact of the digital economy on urban economic resilience, we first conducted a benchmark model, and the results are presented in Table 3. Columns (1) to (5) display the regression results with individual and time effects controlled for under a two-way fixed effect. Specifically, column (1) presents the regression results of digital economy on urban economic resilience without adding any control variable, while columns (2) to (5) show the results when control variables are gradually added.

The regression results in Table 3 provide a preliminarily confirmation that the digital economy has a significant effect on promoting urban economic resilience, supporting H1. Regardless of the inclusion of control variables, the coefficient of digital economy is large, positive, and significant at the 1% level. This suggests that the development of the digital economy can directly improve the resilience of urban economy in three ways: resistance and recovery, adaptation and adjustment, and innovation and development.

The reasons for this may be two-fold: Firstly, cities can use digital industries and technologies to optimize government management and coordination means and empower government services. This can strengthen government risk prevention awareness, improve government governance efficiency, optimize business environment, and promote high-quality social and economic development, thereby improving urban economic resilience. Secondly, digital technology enables cities to quickly match their industrial capacity with market demand, which can improve the overall income of the city, thereby enhancing the resilience of the city’s economy.

Moreover, cities with developed digital economies tend to have greater economic openness. For every 1 unit increase in economic openness, the level of urban economic resilience increases by 0.579 units. Greater economic openness can connect cities with the world, improve the ability of urban economic collaboration and innovation, and add vitality to the innovative development of urban economy.

#### 5.1.2. Heterogeneity Test

To further evaluate the regional heterogeneity of the impact of digital economy on urban economic resilience, this study divided the 258 cities into samples based on city level and geographical location. This approach was used to explore the commercial and regional heterogeneity of the impact of economic development on the improvement of urban economic resilience.

Firstly, the study divided the cities into commercial core and non-core cities based on the “2021 City Business Charm Rankings” compiled by CBN. In doing so, 45 cities involved in first-tier, new first-tier, and second-tier cities were regarded as commercial core cities, and the remaining 213 third-tier, fourth-tier, and fifth-tier cities as non-commercial core cities. The results presented in Table 4 (columns 1 and 2) show that the regression coefficient of the digital economy on the economic resilience of core cities is 0.555 and significant at the 5% level, while the regression coefficient of non-core cities is 0.383 and significant at the 10% level. This indicates the development of digital economy in core cities has a stronger effect on urban economic resilience than in non-core cities. In addition, economic resilience of non-core cities is significantly positively correlated with food security due to the presence of more rural areas that rely on the primary industry.

Second, the study divided the 258 cities into eastern, central and western regions. The results presented in columns 3, 4 and 5 of Table 4 show that the development of digital economy in eastern cities significantly improves urban economic resilience, while its role in central and western regions is not significant. This is because the development cycle, speed, and degree of digital economy of central and western cities are later than eastern cities, and the role of digital economy in improving the economic resilience of non-core cities needs to be further exploited. Moreover, compared with the central and western cities, the eastern cities have higher ownership, utilization and innovation ability of information network technology, and the overall digital divide is smaller. Therefore, in the economic resilience of the eastern cities, the digital economy can play a more promoting role. Lastly, economic openness has the greatest effect on the improvement of economic resilience in eastern regions, while it has no significant effect on the improvement of economic resilience in western regions due to the decreasing degree of foreign investment environment optimization.

#### 5.1.3. Stability Test

In order to more robustly evaluate the enhancement of urban economic resilience by the digital economy, this paper conducts several stability tests on the data. First, since the sample is of “Large N Small T” type, the panel regression does not show the spurious regression phenomenon such as time series models [63] and will give consistent estimates. Therefore, this paper does not carry out stationarity test on the data. However, to robustly evaluate the impact of the digital economy on urban economic resilience, this paper employs dummy variables, shortening the time window, selecting subsamples, and using instrumental variables. The results are as follows.

Firstly, to address the estimation bias introduced by the macro-level changes of individuals over time, this paper introduces province fixed effect and province-year interaction effect, based on Jinhe’s research [41]. The introduction of these dummy variables results in considerable positive effect of digital economy on urban economic resilience, at the 1% level, as shown in Columns 1 and 2 of Table 5. These results are robust to the inclusion of individual macro factors.

Secondly, most studies based on the China Urban Statistical Yearbook use data from 2011 onwards. However, in this study, the sample period is 2004–2017, with missing data supplemented from the city bulletins, provincial statistical yearbooks, and relevant interpolation methods. To avoid the interference from the processed data with the empirical results, the sample is adjusted to 2011–2017, following Weibing [64]. In addition, to mitigate the influence of phased government policies, the sample period is divided into 2004–2011 and 2012–2017, based on the time of the 18th National Congress of the Communist Party of China. Shortening the time window results in a significantly positive effect of digital economy on urban economic resilience at the 1% level, as shown in Columns 3, 4 and 5 of Table 5. These results are robust.

Thirdly, to ensure that the empirical analysis results are not affected by the specific city, the study eliminates the capital city by subsample screening, following Xuenan [65]. Provincial capitals are the cities with the most developed digital economy, the most complete digital infrastructure, the largest concentration of digital talents, and the smallest digital divide in all provinces. The use of sub-samples results in significantly positive effect of digital economy on urban economic resilience, at the 1% level, as shown in Column 6 of Table 5. These results are robust.

Lastly, to address endogenous problem, this paper employs the approach of Zhang [66] by using the product of the spherical distance from the prefecture-level city to the provincial capital city and the year as the instrumental variable for the digital economy development index. The spillover effect of digital economy is expected to increase as prefecture-level cities become closer to the provincial capital city, and the spillover effect of digital economy is expected to increase year by year due to the increasing marginal benefit of digital economy. Moreover, the spherical distance is a natural geographical attribute that weakens urban economic resilience development, making it a stable instrumental variables. Following Xun’s [67] approach, three tests are conducted: over recognition, non-recognition and weak recognition. The results of these tests confirm the rationality of instrumental variables. The impact of digital economy on urban economic resilience remains significantly positive, even after accounting for endogeneity, as shown Table 6 and in the last column of Table 5. These results are robust, indicating the validity of the benchmark regression results.

The four test methods employed in this study provide robust evidence that digital economy has a significant positive impact on urban economic resilience, thus supporting the validity of hypothesis H1.

### 5.2. Further Analysis: Moderated Mediation Effect

To further test how the digital economy enhances the resilience of urban economies, this study analyzes the moderating and mediating effects of mechanism, using a hierarchical regression method. Firstly, stepwise regression is used to test the process with moderated mediation effect, and the results are shown in Table 7. The regression results of Equation (2) reveal that the coefficient $c_{3}$ of the interaction term between carbon emissions and the digital economy is significant, indicating that carbon emissions have a moderating effect on the direct impact of the digital economy on urban economic resilience. The moderated mediation effect of the digital economy on urban economic resilience is studied in terms of IS, PQ and MC.

In the IS-MED regression, the coefficient $a_{1}$ of the digital economy is significant, indicating that the digital economy has a promoting effect on industrial structure that is not moderated by carbon emissions. The coefficient ${c^{\prime }}_{1}$ of the digital economy is significant in the IS-MED regression, indicating that the digital economy has a promoting effect on urban economic resilience that is not moderated by carbon emissions. The coefficient of $b_{2}$, the interaction term between industrial structure and carbon emissions is significant, suggesting that the industry structure has a promoting effect on urban economic resilience moderated by carbon emissions. Due to the non-significant results of the PQ-MED regression, the PQ-RES regression includes only significant DIG, CR, and DIG interaction terms, ${c^{\prime }}_{1},{c^{\prime }}_{3}$, which only indicate that the digital economy has two promoting effects on urban economic resilience, one moderated by carbon emissions and the other not.

In the MC-MED regression, the interaction coefficient $a_{3}$ between carbon emissions and the digital economy is significant, indicating that the digital economy has a restraining effect on above-scale enterprises, which is moderated by carbon emissions.

The coefficients ${c^{\prime }}_{1},{c^{\prime }}_{3}$ of the digital economy and the interaction term of carbon emission and digital economy are significant in the MC-RES regression, indicating that the digital economy has a promoting effect on urban economic resilience, both with and without moderation by carbon emissions. The coefficients $b_{1},b_{2}$ of the interaction term between MC and MC × CR are significant, indicating that above-scale enterprises have a negative effect on urban economic resilience moderated by carbon emissions and a positive effect not moderated by carbon emissions.

To test the validity of the stepwise regression method, the study uses the Bootstrap method to supplement and verify the findings obtained by the coefficient product test on the results of stepwise regression. The confidence interval presented in Table 8 represents the 95% confidence interval that is corrected for bias. A significant coefficient is obtained if 0 is not included in the confidence interval. This indicates that the moderating effect influences the mediating effect. The expressions for the moderating mediating effect and the moderating direct effect are $\left(a_{1}+a_{3}MR\right)\times \left(b_{1}+b_{2}MR\right),\left({c^{\prime }}_{1}+{c^{\prime }}_{3}MR\right)$, respectively. To simplify the expression, this paper considers the moderating variable MR=1, which represents broadcast of the mediating effect and moderating effect.

The findings of the bootstrap tests are presented in Table 8, which indicates the following results: Firstly, the significant paths identified through the stepwise regression method are confirmed by the bootstrap method, indicating the robustness of the obtained results. Secondly, the product test demonstrates that the relationship between digital economy and population quality exhibits a moderated mediation effect. Specifically, the direct effect of digital economy on population quality is facilitated by carbon emissions, while the direct effect of population quality on urban economic resilience is not moderated by carbon emissions. Thirdly, the direct effect test of the three path explanatory variables reveals that the direct effect also has a mediating effect in the “digital economy—industrial structure—urban economic resilience” pathway, despite the lack of significant moderation effect in the stepwise regression method. Lastly, the moderated mediation effects of IS, PQ, and MC are 33.5%, 7.2%, and 19.4%, respectively, and all three test results are significant, indicating no endogeneity problem caused by omitted variables.

**Figure 2:**
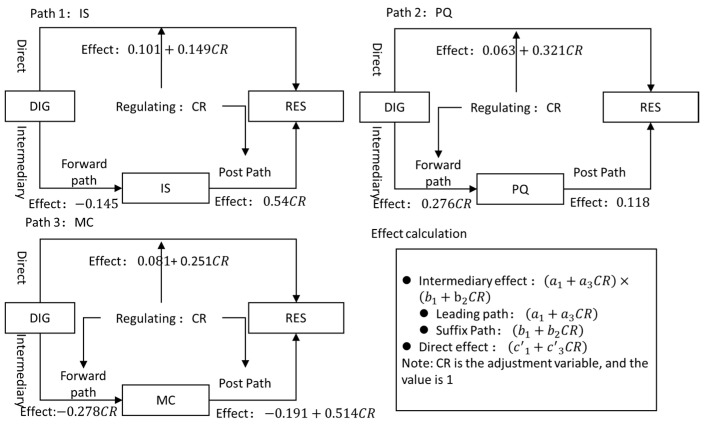
The role of digital economy and carbon emissions on urban economic resilience.

Based on the robustness of the results of both the stepwise regression and Bootstrap tests, the moderating effect of carbon emissions on each pathway is presented in Figure 2, where paths 1, 2 and 3 illustrate the specific effects of IS, PQ and MC, respectively. The upper and lower parts of each path indicate the moderating effects of carbon emissions on direct effects and indirect effects, respectively, while CR represents the carbon emission variable. Overall, the moderating effect of carbon emissions on the direct impact of the digital economy on urban economic resilience is significantly positive. Specifically, in path 1, the moderating effect on indirect effect is post-positive adjustment with a coefficient of 0.54, whereas in path 2, the moderating effect on the indirect effect is front positive adjustment with a coefficient of 0.276. In path 3, the moderating effect on indirect effect is front negative adjustment and post-positive adjustment, with coefficients of −0.278 and 0.514 respectively.

To summarize the moderated mediation effect, the findings are as follows: First, under the role of carbon emissions, the digital economy can enhance the structural stability of primary, secondary and tertiary industries, and indirectly inhibit urban economic resilience, which means H2 does not hold true. Second, under the carbon emission mediating on the front path, the digital economy can improve urban economic resilience by enhancing the quality of urban population, which H3 is established. Third, the digital economy reduces urban economic resilience by diminishing above-scale enterprises under the carbon emission mediating of front and post paths, and, thus, the evidence does not support H4. Finally, besides the mediation effect, the digital economy also has a direct promoting effect on urban economic resilience, which is moderated by carbon emissions, and this supports H5.

This may be due to several reasons. First, although the digital economy can promote the coordinated development of primary, secondary and tertiary industries, it may be more advantageous to develop advantageous industries than to coordinate the development of primary, secondary and tertiary industries for small prefecture-level economies. Especially for some cities with high carbon emissions, the industrial structure of these cities is often dominated by the secondary industry, and in this case, the coordinated development of primary and tertiary industries may have a greater inhibitory effect on the efficiency of urban economic operation. Moreover, extending the industrial chain and optimizing the industrial structure based on prefecture-level cities can easily overlook the regional economic layout and lead to industrial restructuring, thus, wasting urban resources.

Second, on the one hand, the digital economy can improve the overall cultural level of the urban population through digital resources but on the other hand, cities with more developed digital economies provide more comprehensive social public services and more convenient services, which attract more talents to settle in the city, thereby expanding the urban talent team and improving urban economic resilience. In addition, compared with cities with lower carbon emissions, other cities have relatively low environmental comfort, so it is more likely to improve urban comfort through the introduction of digital economy. Therefore, the expansion of digital economy tends to attract more talents to cities with higher carbon emissions.

Third, although the digital economy can lower the threshold of entrepreneurship and thus achieve the goal of “mass entrepreneurship and innovation”, its self-expanding nature will make the Matthew effect more pronounced, thus inhibiting the formation of new above-scale enterprises. Although suppressing above-scale enterprise formation can help alleviate monopolistic behavior of enterprises in order to increase the number of urban enterprises and form a diverse urban economy, thus improving urban economic resilience, it undermines the formation of economies of scale and scope for urban enterprises. Overall, the digital economy’s negative impact on urban economic resilience through its suppression of above-scale enterprises is exacerbated by the effect of carbon emissions.

## 6. Conclusions and Recommendations

This paper examines the impact of digital economy on urban economic resilience by focusing on three key factors: stabilizing industrial structure, introducing talent quality, and forming above-scale enterprises. It puts the digital economy in the same framework as carbon emission and industrial structure, and employs panel data of 258 cities above prefecture level from 2004 to 2017. The study uses a two-way, fixed-effect model and a mediating-moderating model to analyze the sources of enhancement dynamics for urban economic resilience. The findings suggest that the digital economy plays a crucial role in boosting urban economic resilience through its direct impact. This conclusion holds even after various robustness tests at different times and different city sizes. Specifically, the digital economy can stabilize the industrial structure, improve population quality, reduce the formation of large-scale enterprises, and influence the economic resilience of cities under the moderating effect of carbon emissions. Furthermore, the study reveals that the digital economy has a positive promoting effect on the urban economic resilience, while population quality and industrial structure positively effect digital economy. In contrast, scale enterprises negatively impact urban economic resilience. Carbon emissions moderate the relationship between digital economy and urban economic resilience, positively affecting the post path of the digital economy via industrial structure and scale firms, and positively impacting the front path of digital economy via population quality. However, carbon emissions negatively moderate the front path of the digital economy affecting urban economic resilience through scale enterprises. Additionally, individual heterogeneity, including factors such as geographic location, administrative hierarchy, and the city size can affect the extent to which digital economy enhances urban economic resilience. Based on these findings, this paper proposes the following policy recommendations.

Firstly, in order to drive economic growth and development, it is crucial for governments to innovate their urban digital programs and accelerate the growth of the digital economy. This can be achieved by taking “digital industrialization and industrial digitalization” as guidance and implementing urban digital economic action plans. To achieve this, governments should focus on iteratively updating the basic digital infrastructure, such as gigabit networks and 5G communications, and also developing or introducing new emerging digital industries such as artificial intelligence, big data, blockchain, and cloud computing based on local conditions. Additionally, deeper integration of digital applications in the supply chain and marketing can promote the data empowerment of the entire industrial chain transformation.

Secondly, the digital economy should be leveraged to consolidate urban digital capability building and increase the digital economic efficiency of the urban digital capabilities, ultimately leading to the resilient construction of digital cities. This can be achieved by focusing on the deep integration of digital economy, digital technology, and residents’ life, government governance, enterprise management, earth mapping, and other fields. By building the urban basic data base, cities can lay the foundation for the development of digital cities. Electronic governments can be constructed to improve the government’s policy-making level and optimize government service functions. Finally, green digital production should be developed to alleviate dependence on industrial energy. By replacing high-carbon energy such as oil and coal with electrical energy, promoting low-carbon industrial energy, energy-saving production, and clean production, cities can realize sustainable development.

Thirdly, building industrial area layout and realizing industrial synergy development is another crucial step towards economic development. This can be achieved by taking urban agglomerations and urban circles as economic units and leveraging existing industrial foundations and functional positioning in different places to implement differentiated, featured, and synergistic development. By balancing regional and inter-regional industrial relations, industrial chain relations can be formed based on technology and economic correlation, featuring division of labor, cooperation, complementary interaction, and coordination. Governments should also promote the advanced development of the industrial structure by focusing on the breakthrough of basic products and key technologies, and make policies in tiers and categories to solve the bottleneck and short board of industrial chains.

Fourthly, urban digital talents should be cultivated, and the potential of the urban population should be stimulated. To achieve this, governments should optimize the urban living environment and formulate relevant digital talent introduction measures. Efforts should be made to improve the work, policies, services, and social environment for talent development and explore personnel introduction measures from aspects such as urban medical security, convenient travel and transportation, and social welfare benefits. Additionally, importance should be given to digitalization of educational resources and maximizing the potential of talent resources. Governments should take social demand as guidance and market as support, and promote digital education reform in universities. By using digital technology to digitalize learning resources such as vocational education, knowledge training, and popular science education, governments can maximize the permeability and net coverage rate of education.

Finally, to prevent disorderly expansion of capital and expand the scale of market participants, governments should formulate relevant laws and regulations to intervene in unfair competition. This is particularly important to deal with the self-expansion characteristic of digital economy. To promote market fairness and justice, cities should strengthen law enforcement and intervene in the market structure to prevent the emergence of exclusive agreements such as “two choices one”. Additionally, cities should build enterprise incubators, formulate innovation and entrepreneurship supporting policies, and encourage “mass entrepreneurship and innovation”. Innovation and entrepreneurship incubation bases for college students should be established, and entrepreneurship support policies should be implemented to attract graduates to start businesses in their hometowns, enhancing the diversified development of urban economic entities.

## Figures and Tables

**Table 1:** Comprehensive evaluation system of urban economic resilience and digital economy level.

	The Dimension	Indicators	Indicator Description	Attribute
Urban economic resilience	Resistance and resilience	Dependence on foreign trade	Total imports and exports/GDP	−
		Per capita disposable income	Per capita disposable income	+
		Labor productivity of the whole society	GDP/Number of employees in urban units at the end of the period	+
		GDP per capita	GDP/total population at year-end	+
		Share of unemployed population in urban areas	Number of registered unemployed persons in urban areas/total population at year-end	+
	Ability to adapt and adjust	Retail sales of consumer goods per capita	Total retail sales of consumer goods/total population at year-end	+
		Fiscal self-sufficiency rate	Budgeted revenue/budgeted expenditure	+
		Per capita local fiscal expenditure	Budgeted revenue/total population at year-end	+
		Fixed asset investment per capita	Social fixed asset investment/total population at year-end	+
	Innovation and transformation capability	Per capita fiscal expenditure on education	Education expenditure/total population at year-end	+
		Regional innovation and entrepreneurship index	Overall Innovation Index	+
		Advanced industrial structure	Index of advanced industrial structure [59]	+
		Per capita fiscal expenditure on science	Science expenditure/total population at year-end	+
		Scientific research industry employment index	Number of persons employed in scientific research, technical services and geological survey	+
Digital economy level	Digital infrastructure	Internet penetration	Number of Internet broadband access users/total population at year-end	+
		Mobile Internet penetration	Year-end mobile phone users/Number of employees in urban units at the end of the period	+
	Digital industrialization	Internet industry Employment index	Number of employees in information transmission, computer services and software industries/Number of employees in urban units at the end of the period	+
		Postal business revenue	Postal business revenue	+
		Software business revenue	Telecom business revenue	+

**Table 2:** Descriptive statistics of main variables.

Categories	Name	Symbol	Size	Min	Max	Mean	Std
Explanatory	Digital economy	DIG	3612	0.009	0.635	0.082	0.056
Explained	Economic resilience	RES	3612	−0.809	4.424	0.000	0.537
Mediator	Industrial structure	IS	3612	2.866	3.141	2.992	0.074
	Population quality	PQ	3612	343	1311.241	166.865	225.968
	Scale enterprise	MC	3612	0.128	36.346	2.928	3.685
Moderator	Carbon emissions	CR	3612	1.562	129.601	25.388	19.160
Control	Economic density	ED	3612	6.302	116,576.224	2083.547	4927.616
	Population density	PD	3612	4.700	2661.540	437.951	312.676
	Economic openness	EO	3612	20.255	8,602,702.688	409,033.206	800,922.551
	Food security	FS	3612	144.508	402,818.753	3537.549	8190.788

**Table 3:** Results of benchmark regression.

Variable	RES	RES	RES	RES	RES
	1	2	3	4	5
DIG	1.927 ***	0.934 ***	0.876 ***	0.744 ***	0.743 ***
	(0.321)	(0.198)	(0.189)	(0.183)	(0.183)
ED		3.806 ***	4.105 ***	3.882 ***	3.885 ***
		(0.749)	(0.714)	(0.654)	(0.655)
PD			−0.553 ***	−0.533 ***	−0.533 ***
			(0.105)	(0.104)	(0.104)
EO				0.569 ***	0.569 ***
				(0.123)	(0.123)
FS					0.090 ***
					(0.034)
Constant term	−0.579 ***	−0.546 ***	−0.459 ***	−0.468 ***	−0.469 ***
	(0.023)	(0.012)	(0.02)	(0.021)	(0.021)
Individual fixed effects	Yes	Yes	Yes	Yes	Yes
Time fixed effects	Yes	Yes	Yes	Yes	Yes
Number of city	258	258	258	258	258
R^2^	0.87	0.903	0.904	0.908	0.908

Note: *** indicate statistically significant at 1%. Three decimal places are reserved for results. Standard errors are in parentheses.

**Table 4:** Heterogeneity test.

Variable	RES	RES	RES	RES	RES
	Core of Urban	Non-Core Metropolitan	East	Middle	West
DIG	0.555 **	0.383 *	1.307 ***	0.007	0.237
	(0.217)	(0.216)	(0.216)	(0.364)	(0.187)
ED	3.074 ***	9.378 ***	3.386 ***	7.434 ***	9.084 ***
	(0.27)	(1.599)	(0.525)	(2.121)	(2.926)
PD	−0.470 ***	−0.582 ***	−0.373 ***	−0.758 ***	−2.635
	(0.126)	(0.113)	(0.112)	(0.232)	(2.374)
EO	0.371 ***	0.818 *	0.545 ***	0.744 *	−0.158
	(0.115)	(0.42)	(0.089)	(0.448)	(0.221)
FS	−0.077	0.104 **	0.085 **	0.066	−13.597 *
	(0.048)	(0.045)	(0.042)	(2.682)	(6.762)
Constant term	−0.127 **	−0.529 ***	−0.437 ***	−0.474 ***	−0.265
	(0.051)	(0.02)	(0.03)	(0.037)	(0.264)
Individual fixed effects	Yes	Yes	Yes	Yes	Yes
Time fixed effects	Yes	Yes	Yes	Yes	Yes
Number of city	45	213	109	106	43
R^2^	0.965	0.893	0.933	0.893	0.899

Note: ***, **, * indicate statistically significant at 1%, 5%, 10% respectively.

**Table 5:** Robustness tests for regression results.

Variable	Dummy Variable Method		Shortening Time Windows			Subsample	Instrumental
	Provinces Effect	Interaction Effect	Common Sample	Before Eighteenth	After Eighteenth	Non-Provincial Capital	Distance
DIG	0.743 ***	0.684 ***	0.510 ***	0.367 ***	0.517 ***	0.724 ***	2.141 *
	(0.183)	(0.195)	(0.169)	(0.131)	(0.187)	(0.227)	(1.261)
Control	Yes	Yes	Yes	Yes	Yes	Yes	Yes
provinces	Yes	Yes					
Province × Year		Yes					
Instrumental							Yes
Individual fixed	Yes	Yes	Yes	Yes	Yes		Yes
Time fixed	Yes	Yes	Yes	Yes	Yes	Yes	Yes
city	258	258	258	258	258	232	258
period	14	14	7	8	6	14	14
R^2^	0.908	0.941	0.754	0.885	0.673	0.905	0.334 (Centered)

Note: ***, * indicate statistically significant at 1%, 10% respectively.

**Table 6:** Instrumental variables reasonableness test.

Check the Name	The Results of
The first stage	
Underidentification tests	
Anderson can.corr.n * CCEV LM Statistic (Chi-SQ)	14.810 ***
Weak identification test	
Cragg-Donald Wald F statistic	13.730
Anderson-rubin Wald Test (F Test)	3.080 *
Anderson-rubin Wald Test (Chi-SQ)	3.340 *
Stock-wright LM S Statistic (Chi-SQ)	3.330 *
The second stage	
Underidentification test	
Anderson canon. corr. LM statistic	12.545 ***

Note: ***, * indicate statistically significant at 1%, 10% respectively.

**Table 7:** Stepwise regression is used to test the moderated mediation effect.

Variable	Equation (2)	IS		PQ		MC	
	RES	MED	RES	MED	RES	MED	RES
DIG	0.073 * (0.042)	−0.145 ** (0.068)	0.101 ** (0.041)	0.075 (0.066)	0.063 * (0.037)	0.031 (0.044)	0.081 * (0.043)
CR	0.120 * (0.07)	−0.093 (0.088)	−0.279 *** (0.095)	0.035 (0.048)	0.121 (0.079)	0.080 ** (0.039)	0.08 (0.003)
CR × DIG	0.334 ** (0.129)	−0.082 (0.165)	0.149 (0.13)	0.276 (0.182)	0.321 ** (0.137)	−0.278 *** (0.103)	0.251 ** (0.117)
MED			0.015 (0.016)		0.118 ** (0.051)		−0.191 * (0.114)
MED × CR			0.540 *** (0.118)		−0.032 (0.125)		0.514 * (0.293)
Individual effect	Yes						
Time effect	Yes						
Control variables	Yes						

Note: ***, **, * indicate statistically significant at 1%, 5%, 10% respectively.

**Table 8:** Product test of moderated mediation effect of digital economy.

Bootstrap (Resampling Times: 1000)						
Path	IS		PQ		MC	
	Coefficient	Confidence Interval	Coefficient	Confidence Interval	Coefficient	Confidence Interval
$a_{1}\times b_{2}$	−0.078 ** (0.002)	[−0.144, −0.034]	−0.002 (0.000)	[−0.018, 0.004]	0.016 (0.003)	[−0.019, 0.078]
$a_{3}\times b_{1}$	−0.001 (0.000)	[−0.007, 0.001]	0.032 ** (0.000)	[0.010, 0.064]	0.053 ** (0.003)	[0.017, 0.106]
$a_{3}\times b_{2}$	0.044(0.004)	[−0.157, 0.063]	−0.009(0.001)	[−0.052, 0.019]	−0.143 **(0.010)	[−0.331, −0.059]
Moderated mediation effect	−0.126 ** (0.002)	[−0.222, −0.06]	0.03 ** (0.001)	[0.003, 0.065]	−0.08 ** (0.004)	[−0.223, −0.030]
Moderated direction effect	0.25 ** (0.002)	[0.149, 0.379]	0.387 ** (0.000)	[0.272, 0.507]	0.332 ** (0.003)	[0.241, 0.432]
Proportion of mediation effect	33.5%		7.2%		19.4%	
Individual effect	Yes					
Time effect	Yes					
Control variables	Yes					

Note: ** indicate statistically significant at 5%.

## Data Availability

The data used to support the findings of this study are included within the paper. The carbon emission data are available from the China Carbon Emission Accounts & Datasets (CEADs), and the innovation and entrepreneurship index was obtained from Center for Enterprise Research of Peking University. The rest of the data are obtainable from China Urban Statistical Yearbook, provincial Statistical Yearbooks, some city Statistical bulletins, China Customs, EPS database, and National Research Network.
